# Targeting Src-Hic-5 Signal Cascade for Preventing Migration of Cholangiocarcinoma Cell HuCCT1

**DOI:** 10.3390/biomedicines10051022

**Published:** 2022-04-28

**Authors:** Wen-Sheng Wu, Chin-Hsien Ling, Ming-Che Lee, Chuan-Chu Cheng, Rui-Fang Chen, Chen-Fang Lin, Ren-In You, Yen-Cheng Chen

**Affiliations:** 1 Division of General Surgery, Department of Surgery, Hualien Tzu Chi Hospital, Buddhist Tzu Chi Medical Foundation, Hualien 970, Taiwan; wuws@gms.tcu.edu.tw (W.-S.W.); lcs0730@tzuchi.com.tw (C.-H.L.); cordiallove@yahoo.com.tw (C.-C.C.); 106721106@gms.tcu.edu.tw (R.-F.C.); chenfang0223@gmail.com (C.-F.L.); 2 Institute of Medical Sciences, Tzu Chi University, Hualien 970, Taiwan; 3 Division of General Surgery, Department of Surgery, Wan Fang Hospital, Taipei Medical University, Taipei 110, Taiwan; mclee1229@mail.tcu.edu.tw; 4 Department of Surgery, School of Medicine, College of Medicine, Taipei Medical University, Taipei 110, Taiwan; 5 Department of Laboratory Medicine and Biotechnology, College of Medicine, Tzu Chi University, Hualien 970, Taiwan; yri100@mail.tcu.edu.tw; 6 School of Medicine, Tzu Chi University, Hualien 970, Taiwan

**Keywords:** cholangiocarcinoma, hydrogen peroxide clone-5, nonreceptor tyrosine kinase Src, HuCCT1, migration assays

## Abstract

Cholangiocarcinoma (CCA) is the second most common primary liver cancer with poor prognosis. The deregulation of a lot of oncogenic signaling molecules, such as receptor tyrosine kinases (RTKs), has been found to be associated with CCA progression. However, RTKs-based target therapy showed limited improvement suggesting a need to search for alternative targets for preventing CCA progression. To address this issue, we screened the oncogenic signal molecules upregulated in surgical tissues of CCAs. Interestingly, over-expression of hydrogen peroxide inducible clone-5 (Hic-5) coupled with over-activation of Src, AKT, JNK were observed in 50% of the cholangiocarcinoma with metastatic potential. To investigate whether these molecules may work together to trigger metastatic signaling, their up-and-down relationship was examined in a well-established cholangiocarcinoma cell line, HuCCT1. Src inhibitors PP1 (IC50, 13.4 μM) and dasatinib (IC50, 0.1 μM) significantly decreased both phosphorylated AKT (phosphor-AKT Thr450) and Hic-5 in HuCCT1. In addition, a knockdown of Hic-5 effectively suppressed activation of Src, JNK, and AKT. These implicated a positive cross-talk occurred between Hic-5 and Src for triggering AKT activation. Further, depletion of Hic-5 and inhibition of Src suppressed HuccT1 cell migration in a dose-dependent manner. Remarkably, prior transfection of Hic-5 siRNA for 24 h followed by treatment with PP1 or dasatinib for 24 h resulted in additive suppression of HuCCT1 migration. This suggested that a promising combinatory efficacy can be achieved by depletion of Hic-5 coupled with inhibition of Src. In the future, target therapy against CCA progression by co-targeting Hic-5 and Src may be successfully developed in vivo.

## 1. Introduction

Cholangiocarcinoma (CCA) is the second most common primary liver cancer [1] and the overall incidence of CCA has increased progressively worldwide [2]. CCA originates from the epithelium lining the biliary tree and can be divided into two main classes in accordance with the anatomical origin: intrahepatic CCA (iCCA) and extrahepatic CCA (eCCA) [3]. eCCA is further distinguished into perihilar CCA (pCCA) and distal CCA (dCCA) with the cystic duct between them.

CCA is an aggressive tumor, and most patients are diagnosed upon tumors entering late stage with a median survival of less than 24 months. Surgery is effective in early stages whereas chemotherapy (5-fluoruracil, gemcitabine, oxaliplatin, etc.) is required for surgery of locally advanced CCA. For patients with advanced-stage or unresectable CCA, the current systemic therapies are of limited effectiveness and the median overall survival with the treatment of gemcitabine and cisplatin is less than one year. Thus it is urgent to develop effective therapeutic strategies to improve the prognosis of CCA. Among them, the molecule-based-targeted therapies against metastasis of CCA are promising. Tumor metastasis is initiated by epithelial mesenchymal transition (EMT), migration, and invasion followed by intravasation, extravasation, and final invasion on metastatic loci [4,5]. Molecular mechanisms for CCA progression have been investigated in the past decades. Specifically, over activation of oncogenic signaling pathways involved in CCA progression were emerging in recent years, among them the receptor tyrosine kinase (RTK)-mediated pathways were highlighted. The deregulations of a lot RTKs such as EGFR and HER2/neu, VEGF, PDGF, and FGFR2 have been found to be associated with CCA progression [6]. For example, EGFR and HER2/neu overexpression have been detected in 10–32% of iCCA [7,8], and VEGF was found to overexpress in 53.8% of iCCAs and 59.2% of extrahepatic CCAs. In addition, chromosomal fusions of FGFR2 with multiple genomic partners led to its activation in iCCA [9]. Thus, a lot of RTK inhibitors have been employed for target therapy of CCA. These include erlotinib (EGFR tyrosine kinase inhibitor), cetuximab (an anti-EGFR antibody), and cabozantinib (a recombinant anti-monoclonal antibody) for CCAs with deregulated EGFR, HER2, and c-Met, respectively. However, most of these trials show limited or no improvement [6]. For example, although promising preclinical results have been obtained with ErbB-targeting agents in suppressing cholangiocarcinoma cells both in vitro and in vivo, current clinical ErbB-directed therapies demonstrated only very limited anti-tumor activity [8]. This might largely be ascribed to the complex interactive signaling between ErB and other RTKs that cause drug resistance with single ErbB inhibition (for review, [8]). Similarly, discouraging results have been obtained in several phase II studies where sorafenib and lapatinib (multi-tyrosine kinase inhibitors) have been used as monotherapy (for review, [7]). Although combined RTKs targeting has been suggested for more comprehensive therapeutic approach (for review, [7,8]), the risk of causing severe toxic side effects using more RTK inhibitors (TKIs) is another concern. Therefore, it is an unmet need to search for more novel targets other than RTKs for preventing CCA progression more effectively and safely.

Another issue for target therapy of CCA involves precision medicine. There are various subsets of CCAs with heterogeneous molecular alterations leading to deregulation of distinct signaling cascades. Due to this molecular complexity, it is not surprising that previous targeted therapy against CCA have no satisfactory results [6]. Therefore, the deregulated signal molecules in each individual CCA patient must be comprehensively identified for more precise and personalized target therapy using suitable antagonists.

In this study, we screened the deregulated signaling in the surgical tissues of CCA patients and found the activities and/or expression of several oncogenic molecules downstream of RTKs, including hydrogen peroxide clone5 (Hic-5), Src, AKT, and JNK known to be essential for metastasis, elevated in tissues of a significant portion of metastatic CCA. These molecules are within the same signal cascade in HuCCT1, a conventionally used CCA cell line. Inhibitory study and siRNA technique further validated the Hic-5-Src cascade as a target for preventing HuCCT1 migration.

## 2. Materials and Methods

### 2.1. Collection of CCA Tissues

CCA tissues were collected during surgery at Tzu Chi Hospital, Hualien, Taiwan with patient’s consent, approved by the Research Ethics Committee in Buddhist Tzu Chi General Hospital (IRB 109-148-A). The tissues were snap-frozen at −80 °C before being harvested for Western blotting or sectioning for immunohistochemical (IHC) analysis.

### 2.2. Cell Culture

HuccT 1 is a human bile duct carcinoma cell line with metastatic ability, originally obtained from Cell Bank in RIKEN BioResource Research Center (Ibaraki, Japan) (No. RCB1960). The cells were cultured in RPMI1640 + 10% FBS kept at 37 °C, 5% CO_2_.

### 2.3. Antibodies and Chemicals

Rabbit polyclonal antibodies of Hic-5, p-Src, p-AKT, and AKT were purchased from GeneTex (Irvine, CA, USA) whereas mouse monoclonal antibodies of EGFR, c-Met, Her2, Her3, p-JNK, JNK and rabbit polyclonal antibody of c-Src were from Santa Cruz Biotechnology, Inc. (Dallas, TX, USA). Src kinase inhibitors PP1 were from Merck (Darmstadt, Germany) and dasatinib was from Tocris Bioscience (Bristol, UK).

### 2.4. RNA Interference

Hic-5 expression was transiently knocked down by 12–25 nM Hic-5 siRNA (Dharmacon, Lafayette, CO, USA) for 48 h, according to the manufacturer’s protocol. The depletion of Hic-5 was validated by Western blot and RT-PCR. The target sequence of Hic-5 siRNA is “GGAGCUGGAUAGACUGAUG”.

### 2.5. Proliferation Assay

Cell proliferation assay was performed by utilizing 3-[4,5-dimethylthiazol-2-yl]-2,5-diphenyl tetrazolium bromide (MTT) (Sigma-Aldrich, Poole, UK) as a water-soluble yellow dye that is readily taken up by viable cells and reduced by the action of mitochondrial dehydrogenases. After appropriate treatment of the cells, the dye was released and dissolved by DMSO and the absorbances were measured under 535 nm wavelength.

### 2.6. Wound Healing Migration Assay

A wound healing migration assay was performed using a culture insert (ibidi GmbH, Gräfelfing, MUC, Germany) with a septum located between two small chambers for cell seeding. After cells reached confluence in complete medium for 24 h, the culture insert was removed followed by monitoring the migration of cells toward the blanking area between two monolayers of cells within 48 h of appropriate treatments under serum-free condition. Quantitation of cell motility was performed by counting the cells that have migrated into the blanking area using Image J software (version 1.50 i).

### 2.7. Transwell Migration Assay

Cells were seeded on a 24-well trans well migration insert (Nalge Nunc International, Rochester, NY, USA) in a complete medium for 24 h. After appropriate treatments, cells that had migrated to the underside of the insert membrane were stained with 0.3% crystal violet. The cells on the topside of the insert membrane were rubbed with a cotton swab. The migrated cells on the underside were imaged using phase-contrast microscopy with ×200 magnification. Quantitation of the migrated cell was performed by measuring the intensity of crystal violet staining, using Image J software.

### 2.8. Immunohistochemistry (IHC)

IHC was performed by the EnVision+ Dual Link System-HRP (DAKO, Carpinteria, CA, USA), a two-step staining technique using an HRP labeled polymer conjugated with secondary antibodies. Briefly, the tissue section was incubated with Dual Endogenous Enzyme Block to remove any endogenous peroxidase activity. Subsequently, the sample was incubated with primary Ab for 30 min, followed by the second Ab-HRP labeled polymer for 30 min. Staining is completed by a 5–10 min incubation with 3,3′-diaminobenzidine (DAB+) substrate chromogen which results in a brown-colored precipitate at the antigen site.

### 2.9. Statistical Analysis

ANOVA test was conducted to analyze the intensity differences between samples on the Western blot and the differences in cell motility between the indicated CCAs. Quantitative data were expressed as mean ± coefficient variation (CV). Correlation analyses assessing the expression and/or activation of the signal molecules associated with the extent of metastatic potentials of various CCAs were analyzed by a Chi-square test (SPSS 16.0 software, Chicago, IL, USA).

## 3. Results

### 3.1. Screening the Enhanced Signal Molecules Associated with CCA Metastasis

**Figure 1:**
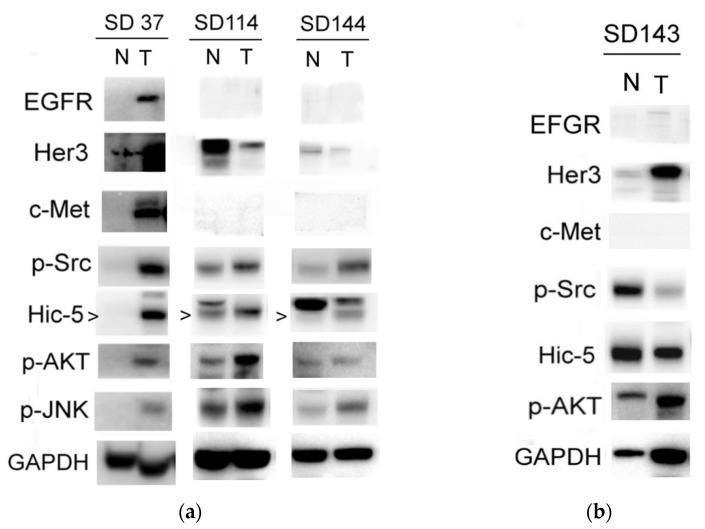
Signal molecules deregulated in CCA tissues. Western blots of indicated molecules in tumor (T) and normal counterpart (N) from different CCA tissues of patient SD37, SD114, SD 144 (**a**), and SD143 (**b**) were performed, GAPDH is included as an internal control. The intensities of each signal molecule in the tumor and normal counterpart are compared using image J software. The data were representative of two reproducible results.

**Figure 2:**
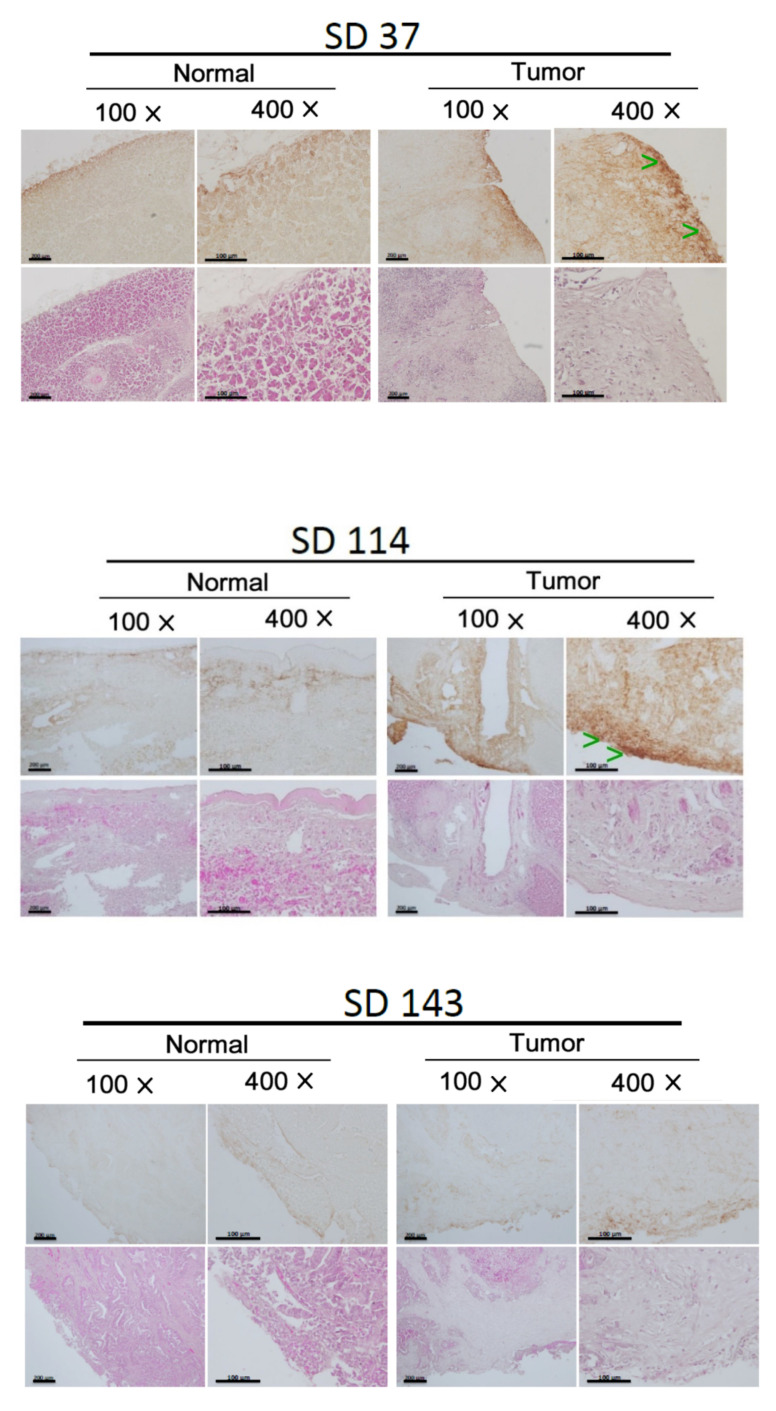

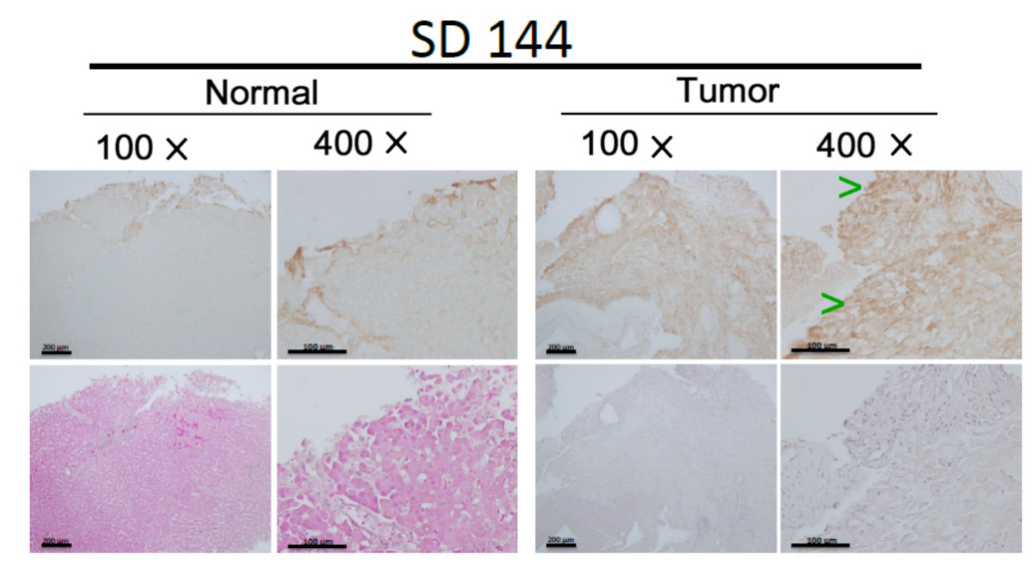
IHC of Hic-5 on tumor and normal counterpart of CCA.

To identify the signal molecules as potential therapeutic targets for preventing CCA progression, about 15 surgical tumor tissues of CCA were obtained from Hualein Tzu Chi hospital (IRB 109-148-A). The samples were processed for Western blot of a lot of critical signal molecules known to be involved in tumor metastasis of CCA, including the oncogenic RTKs (EGFR, Her2, Her3, and c-Met) [10,11,12]; Src, one of the nonreceptor tyrosine kinase [13,14]; JNK, one of the MAPK [15,16]; and AKT, a downstream molecule of PI3K [17,18,19,20]. In addition, Hic-5, one of the focal adhesion molecules known to be associated with progression of hepatocellular carcinoma (HCC) [21,22,23], was included. More than a two-fold increase of the expression and/or activation of the aforementioned signal molecules in tumor tissues compared with those in the normal counterparts were regarded as over-express or over-activated. Conversely, for the association of the signal molecules elevated in tumor tissues with metastasis, clinical-pathological data of CCA patients were employed. The criteria for evaluation of the metastatic potentials of each CCA tumor were set as 0, +, ++, +++ according to whether tumor cells were present in the lymph-vascular system and the number of lymph nodes exhibiting tumor invasion (Table 1). Among the CCA tested, 90% of them exhibited minor (+), moderate (++) or high (+++) metastatic potentials (Chi-square test, *p* < 0.05, *n* = 15). After Western blot analysis of the signaling molecules in CCA tissues, correlation analyses were performed to assess whether the over-expression and/or over-activation of the aforementioned signal molecules were associated with the extent of metastatic potentials of various CCAs. Accordingly, Hic-5 proteins were significantly increased in about 65% of metastatic CCA tissues (Chi-square test, *p* < 0.05, *n* = 13) similar to that observed in HCC [10]. Remarkably, over-expression of Hic-5 coupled with over-activation of Src, AKT, JNK (but not multiple RTKs such as EGFR, c-Met, Her2, and Her3) were observed in 50% of the metastatic CCAs (Chi-square test, *p* < 0.05, *n* = 13). In contrast, only 10–20% of them exhibited overexpression of multiple RTKs such as EGFR, c-Met, and Her3 but not elevated Hic-5, p-Src, p-AKT, and p-JNK (data not shown). In addition, 15% of CCA did not show any increase of these signal molecules. Representative results of four of the metastatic CCA samples SD37, SD114, SD143, and SD144 are demonstrated in Figure 1. Case SD37, SD114, and SD144 (Figure 1a) exhibited simultaneous over-expression of Hic-5 coupled with over-activation of Src, AKT, JNK, compared with that in the normal counterpart. In contrast, EGFR and Her3 but not Hic-5, p-Src, p-AKT, and p-JNK, were elevated in SD143 (Figure 1b). We further validated the expression of Hic-5 on CCA tissue by immunohistochemistry (IHC) analysis. As demonstrated in Figure 2, much higher Hic-5 staining was observed in tumor tissues of SD37, SD114, and SD144 (but not SD143) than that in the normal counterpart, consistent with those observed in Western blot (Figure 1a,b). Interestingly, most of the enhanced Hic-5 staining locates within the peripheral regions which can be suspected of the migration front of the primary tumor.

IHC of Hic-5 on tissue section in CCA (tumor) and normal counterpart (normal). H. *E. stains* for each sample are included on the low panels of each figure. Green arrowheads indicate the locations of high Hic-5 staining (brown color) in the peripheral region of each tumor tissue, photographed under 100- and 400-fold of magnification.

### 3.2. Cross Talk between Hic-5 and Src for Activating AKT in HuccT1

**Figure 3:**
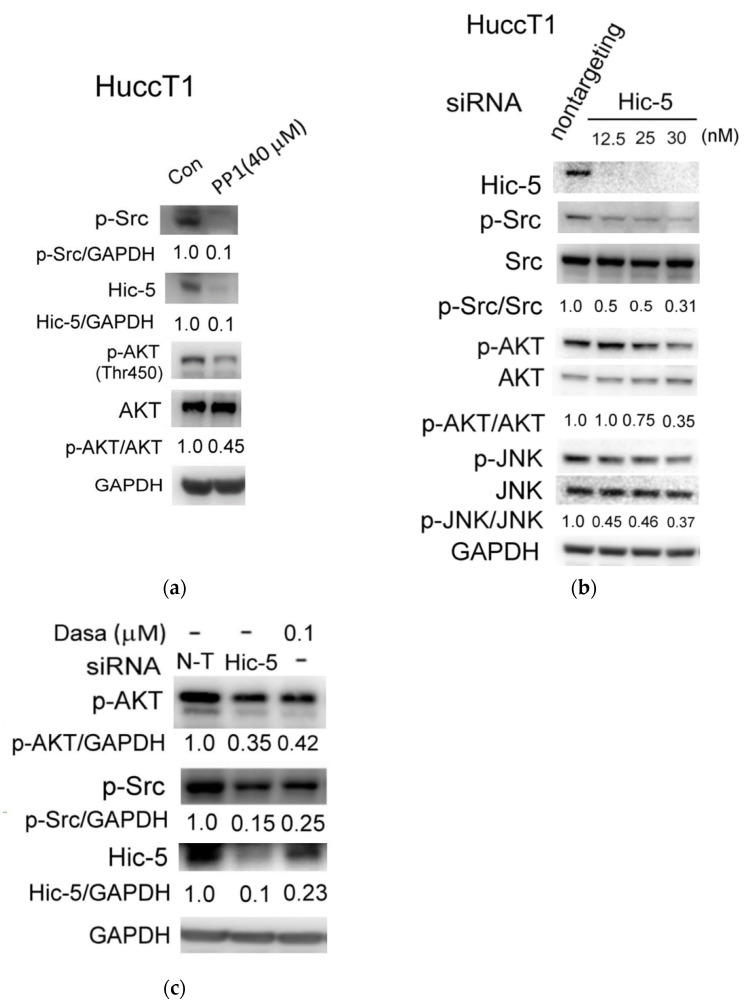
Delineating Src-Hic-5-AKT/JNK signaling in HuCCT1. HuCCT1 cells were treated with Src inhibitor (PP1) (**a**) and Dasatinib (Dasa) (**c**) at indicated concentration; HuCCT1 cells were transfected with Hic-5 siRNA at indicated concentration for 48 h (**b**). Western blot of indicated molecules were performed using GAPDH as an internal control. The numbers below the indicated blots were the relative intensity of each active signaling molecule normalized with their inactive forms or GAPDH as indicated. The data shown are averages of two reproducible results with coefficient of variation (C.V.) as 7.0%.

It is worthy of noting that not only the roles of Hic-5, Src, AKT, and JNK in metastatic signaling have been well established as mentioned above, but also the interaction and regulation between them were frequently mentioned. For example, Src activity was required for Hic-5 to promote TGF-β induced EMT, AKT-mediated EMT [24], and migration of gastric cancer [25]. In addition, JNK positively cross-talks with Hic-5 in the signaling pathway leading to HCC progression [22]. Since we found a large proportion of the metastatic CCAs exhibited simultaneous over-expression of Hic-5 and over-activation of Src, AKT, and JNK, it is very probable that they work together to trigger metastatic signaling for CCA progression. To address this issue, the up-and-down relationship of them were examined in a well-established CCA cell line, HuCCT1. HuCCT1 is derived from ascites of intrahepatic CCA (NCIt:C35417) within the metastatic site (ORDO: Orphanet 70567). Specifically, over-expression of Hic-5 coupled with activation of Src, AKT, and JNK were observed in HuCCT1 (data not shown). To begin with, we found one of the Src inhibitors PP1 (an inhibitor of the Src family Lck/Fyn) at 40 μM concentration dramatically suppressed protein expression of Hic-5 (by 90%) and significantly inhibited the activation of AKT (represented by phosphorylated AKT at Thr450) by 55% (Figure 3a). Moreover, a highly potent Src inhibitor (dasatinib) at 0.1 μM greatly inhibited the expression of Hic-5 and activation AKT by 77 and 58%, respectively (Figure 3c). However, phosphorylation of JNK (p-JNK) was not significantly inhibited by both Src inhibitors (data not shown). Conversely, knockdown of Hic-5 greatly suppressed the activation of Src (represented by phosphorylated Src at Tyr416, p-Src) and phosphorylation of JNK by 50–69% in a dose-dependent manner using 12.5, 25, and 30 nM Hic-5 siRNA (Figure 3b), whereas AKT phosphorylation was significantly inhibited by Hic-5 siRNA at 25 and 30 nM (by 25–65%) but not at 12.5 nM (Figure 3b). Thus, Hic-5 expression was required for activation of Src, and on the contrary, Src activity was also required for Hic-5 expression indicating Hic-5 locates both upstream and downstream of Src. Whereas both Hic-5 and Src were required for AKT phosphorylation, Hic-5 but not Src was required for JNK phosphorylation. Taken together, these results implicated signal cross-talk occurred between Hic-5 and Src to trigger AKT activation in HuCCT1. However, JNK seems located downstream of Hic-5 but not Src and probably not involved in the signaling pathway regulated by Src-Hic-5 cascade.

### 3.3. Depleting Hic-5 Expression and Inhibiting Src Activity Suppress HuccT1 Migration in a Concerted Manner

**Figure 4:**
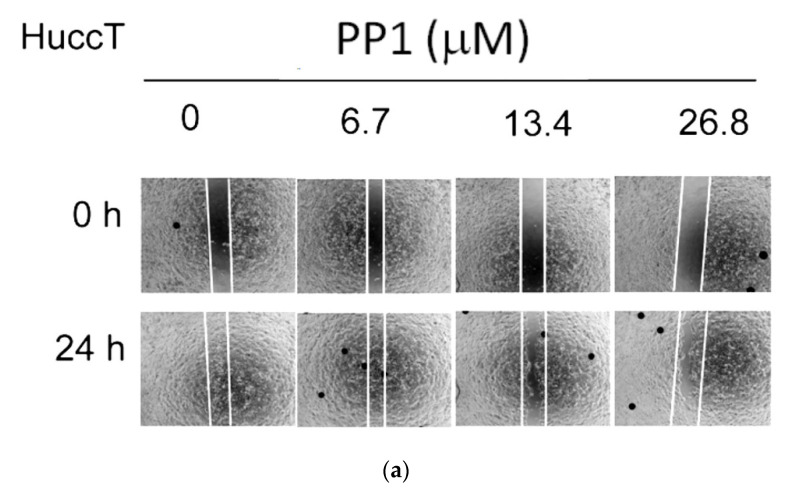

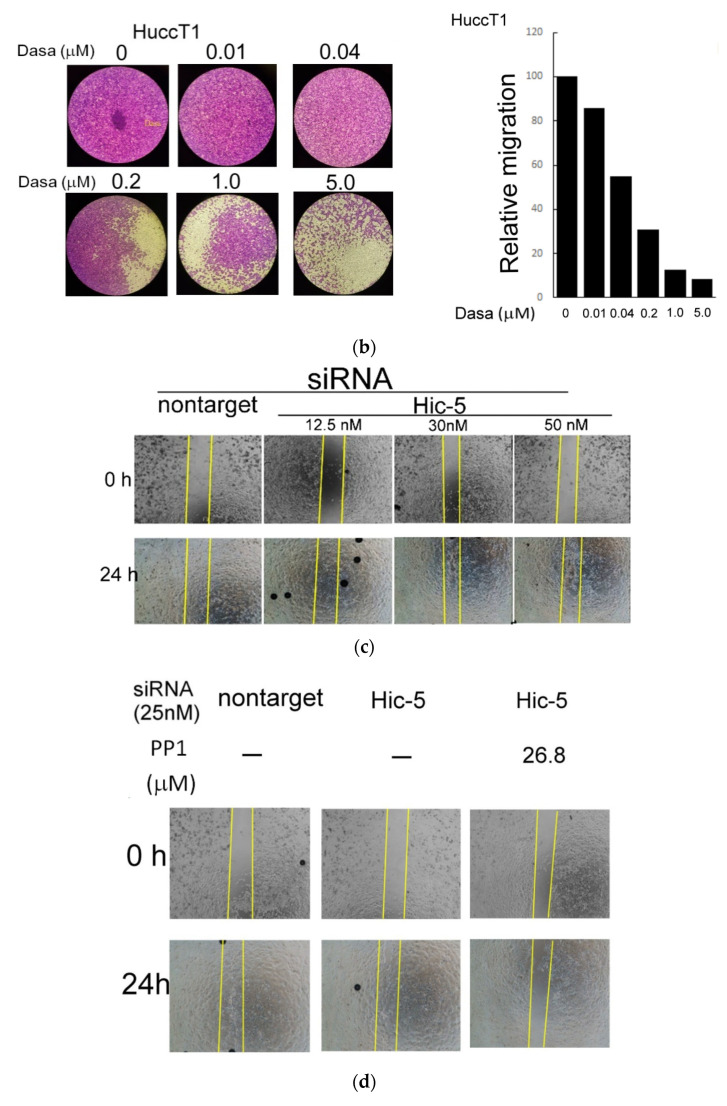

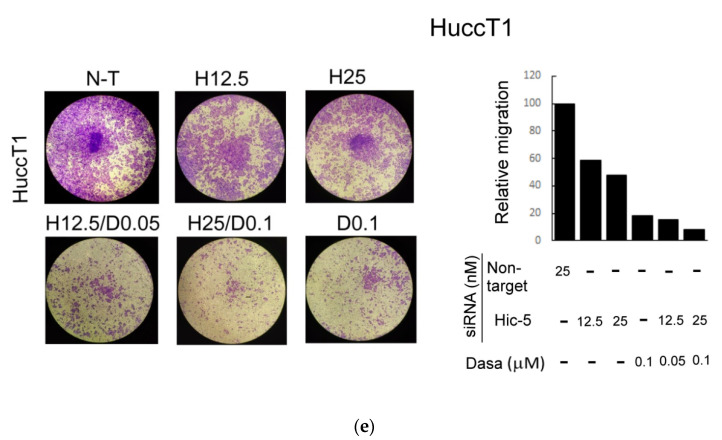
Src-Hic-5 signal cascade is essential for HuCCT cell migration. HuCCT1 cells were pre-treated with Src inhibitor PP1 (**a**) and dasatinib (**b**) at indicated concentrations for 24 h (**a**); HuCCT1 were transfected with Hic-5 siRNA at the indicated concentration for 24 h (**c**); HuCCT1 were transfected with Hic-5 siRNA for 24 h at the indicated concentration, followed by pre-treatment with Src inhibitor PP1 (**d**), and dasatinib (**e**) at the indicated concentration for 24 h. Wound healing culture (**a**,**c**,**d**) and trans well migration assay (**b**,**e**) were performed. In wound healing assays (**a**,**c**,**d**), pictures were taken at 0 and 24 h after the cells begin to move into the wound area between lines. The motility of the cells was compared by the difference of the cell migrated into the wound area at 24 h. In trans well migration assay (**b**,**e**) pictures were taken after dasatinib treatment for 24 h (**b**) or transfection with Hic-5 siRNA for 24 h followed by treatment with dasatinib for 24 h (**e**). Quantitative data of (**b**,**e**) for estimating the intensity of migrated cells on each subfigure using Image J. software were shown in the right panel of each figure. Relative migrations were calculated taking the data of vehicle (**b**) or nontargeting (N-T)siRNA group (**e**) as 100%. The data shown were averages of two reproducible results with C.V. as 10%. In (**b**), Dasa: dasatinib, In (**e**), N-T: nontargeting, H: Hic-5siRNA (nM), D: dasa (μM).

We further investigated whether the Hic-5-Src positive feedback signal cascade is responsible for the tumor progression of HuCCT1. Initially, we examined whether Hic-5-Src signaling is required for cell growth of HuCCT1 using an MTT assay. The results showed that inhibition of Src activity by dasatinib (at 0.01–0.2 μM) but not knockdown of Hic-5 (using Hic-5 siRNA at 25 nM) marginally inhibit cell proliferation (by about 8–10%) of HuCCT1 (data not shown). Thus this signal cascade is not majorly involved in the proliferation of HuCCT1. Further, we examined whether Src activity is required for HuCCT1 cell migration by wound healing assay using Src inhibitor PP1. The cells were pre-treated with various doses of PP1 followed by re-plating the inhibitor-treated cells into a wound healing culture insert. As shown in Figure 4a, PP1 (6.7~26.8 μM) suppressed HuCCT1 cell migration by 30–70%. Further, using the trans well assay, HuCCT1 cell migration was effectively suppressed by the more potent Src inhibitor, dasatinib (at 0.01 to 5 μM), by 14–90% in a dose-dependent manner (Figure 4b). Thus Src activity is essential for HuCCT1 cell migration. To assess whether Hic-5 expression is also required for the migratory ability of HuCCT1, we transfected the cell with different doses (12, 30, and 50 nM) of Hic-5 siRNA, followed by observing the extent of decrease in cell motility using a wound-healing assay. As demonstrated in Figure 4c, cell motility was decreased by 30–45% in cells transfected with 30 and 50 nM Hic-5 siRNA and marginally suppressed by 12.5 nM Hic-5 siRNA. This indicated depletion of Hic-5 decreased HuCCT1 cell migration in a dose-dependent manner. Since we found positive cross-talk between Hic-5 and Src (Figure 3a,b), we investigated whether targeting both Src and Hic-5 simultaneously may augment the suppressive effect on cell motility. As demonstrated in Figure 4d, prior transfection with Hic-5 siRNA (25 nM) for 48 h suppressed HuCCT1 migration by about 25% compared with the non-targeting siRNA group. Interestingly, prior transfection of Hic-5 siRNA (25 nM) for 24 h followed by treatment with 26.8 uM PP1 for 24 h resulted in 100% suppression of HuCCT1 migration (Figure 4d, first panel from right). Moreover, in a trans well migration assay 12.5 and 25 nM Hic-5 siRNA suppress HuCCT1 migration by 40–50% whereas 0.1 μM dasatinib exhibited an 82% suppressive effect (Figure 4e). Remarkably, Hic-5 siRNA (12.5 nM) coupled with 0.05 μM dasatinib exerted a greatly suppressive effect on HuCCT1 cell migration (by 85%), not only much higher than that of 12.5 or 25 nM Hic-5 siRNA but also comparable to that of 0.1 μM dasatinib alone. This indicated that transfection of Hic-5 siRNA at a lower amount may lower the concentration of dasatinib required for effective suppression of cell migration. Moreover, 25 nM Hic-5 siRNA coupled with 0.1 μM dasatinib almost abolished HuCCT1 cell migration (by 92%). Taken together, we suggested that Hic-5 and Src positively cross-talk with each other to induce the downstream AKT triggering HuCCT1 migration in a collaborative manner.

## 4. Discussion

### 4.1. The Role of Focal Adhesion Signaling Molecules Responsible for Cell Migration in Tumor Metastasis

It has been well established that cell migration is one of the critical steps in tumor metastasis [26,27,28,29]. Cell migration is triggered by signaling pathways not only from cellular receptors (such as RTK) but also from focal adhesion signaling molecules including integrin, focal adhesion kinase (FAK), Src, Rac [30], Hic-5 [23,24], and other integrin-associated proteins. In one early study, IHC demonstrated Ep-CAM, an integrin-associated focal adhesion molecule capable of regulating cancer cell adhesion [31], was overexpressed in 95% CCA but not HCC [32]. FAK activation mediated HGF-induced proliferation and invasion of the human cholangiocarcinoma cell line, HuCCA-1 [33]. This was essential for tumor necrosis factor-alpha-dependent matrix metalloproteinase-9 production in a CCA cell line, CCKS1 [34]. A recent study further demonstrated FAK activation contributes to the initiation and progression of iCCA by inducing the YAP proto-oncogene [35]. Regarding the focal adhesion molecules in our study, not only that Src [30] and Hic-5 [21,22] are key effectors responsible for cell migration and tumor progression but also they may interact with each other [36]. Thus, targeting critical signal complex in focal adhesion may prevent metastasis. For example, it is promising to target Rac [37] and integrin [38] for preventing the progression of solid tumor such as cutaneous melanoma. Herein we suggested Src and Hic-5 can be candidate targets for therapeutic approaches against the progression of CCA.

### 4.2. Targeting Hic-5 and Src Is Promising in Preventing CCA Progression

In the past decades, the local and systemic therapies along with target therapy aiming at metastatic signaling cascade including various oncogenic RTKs and diverse intracellular signaling did not significantly improve the prognosis of CCA patients, and strongly suggests the need for novel therapeutic agents and strategies. One of the major obstacles in targeting RTK signaling is due to acquired or inducible drug resistances. Usually, targeting upstream signal molecules is often attenuated by downstream or parallel alterations in the pathway. This is exemplified by resistance to anti-EGFR therapies in colon cancer, which can be mediated by downstream KRAS- or NRAS-activating mutations. In addition, RTK target therapy may be useless due to co-expression of multiple growth factors that can raise compensatory secondary signaling after treatment with specific tyrosine kinase inhibitors (TKIs) [39,40]. For example, in the anti-EGFR target therapy using gefitinib for lung cancer, MET (receptor of HGF) amplification leads to resistance by activating ERBB3 [41] signaling. Recently, metastatic signaling downstream of oncogenic receptors including Hic-5 and Src are emerging. Previously, Hic-5 is increased upon activation of 20-HETE/GPR75 (G-protein coupled receptor 75) that triggering metastatic features of androgen-insensitive prostate cancer cells [42]. In addition, Hic-5 is one of the critical mediators for c-Met to trigger HCC progression [43] and regulated ESCC cell migration and invasion induced by TGFβ [44]. Conversely, Src kinase is one of the downstream signal molecules mediating HER3 (coupled with EGFR or HER2) triggered tumor progression [45]. In addition, Src mediated the reactivation of RTK signaling responsible for the induced resistance of BRAF and MEK inhibitors during the treatment of BRAF mutant melanoma [46]. In the past decades, Src has been found to be a promising target for suppressing tumor progression. For example, an innate immune sensor, NOD1, exerted its antitumor effect on HCC by directly inhibiting the Src-MAPK axis. [47]. Moreover, dasatinib prevented CCA progression in PDX models [48] and reduced the viability of sorafenib-resistant (SR) HCC cells by inhibiting Src [49]. In this report, inhibition of Src activity by dasatinib (at 0.1 μM) prevented cell migration of HuCCT1 by 80% (Figure 3), and suppressed HuCCT1 cell proliferation by 8–10% (data not shown), consistent with the inhibitory effect of dasatinib on CCA progression observed in vivo [48]. Conversely, whether Hic-5 can be a target for preventing metastasis of tumors are far less investigated in preclinical/clinical setting. Previously, siRNA silencing of Hic-5 suppressed migration and invasion of pancreatic tumor [50] and HCC [21,22]. Herein, we demonstrated knockdown of Hic-5 suppresses migration of HuCCT1 in a dose-dependent manner (Figure 4c). It is worth investigating whether depletion of Hic-5 may prevent CCA progression in vivo.

### 4.3. Combined Therapy Is More Effective in Blocking Metastatic Signaling

Recently, combined therapy is emerging as a more effective and safe therapeutic approach in cancer prevention. This is based on multiple targeting against the metastatic pathways may reduce the compensatory signaling encountered in single targeting approaches. Moreover, a lower concentration of each of the inhibitors used in combined treatment can avoid the side effects caused by drugs at higher concentrations used in single drug treatments. In fact, co-targeting PARP1 and Src have been found to improve the therapeutic strategies for HCC [51]. In the present study, we demonstrated co-targeting Hic-5 and Src, which positively crosstalk with each other, may enhance the inhibitory effect on migration of HuCCT1 under lower concentration of Hic-5 siRNA and Src inhibitor dasatinib (Figure 4e).

## 5. Conclusions and Perspective

To develop more precise and personalized therapeutic strategies for improving the prognosis of CCA, the potential molecular target associated with metastasis of CCA were identified in CCA tissues. Elevated expression of Hic-5 coupled with activation of Src and the downstream AKT and JNK were observed in 50% metastatic CCAs. Pathway analysis further proved that Hic-5 and Src, which are critical interactive proteins in focal adhesion, cross-talk with each other to trigger the downstream AKT signaling. Remarkably, simultaneous suppression of both Hic-5 expression and Src activation prevent HuCCT1 migration in a collaborated manner. Although cell migration is one of the essential steps in tumor metastasis, the therapeutic implication conveyed in this study needs to be validated in more advanced models, such as 3D cultures using animal [52] and human [53] tissues. These can be employed for validating the efficiency of the double Hic-5/Src targeting approach in preventing the progression of CCAs with enhanced Hic-5-Src signaling.

## Figures and Tables

**Table 1:** Criteria for evaluation of Metastatic potential.

	Metastatic Potential			
**Metastatic Index**	**+++ ^a^**	**++**	+	0
Lympho/vascular invasion	∨ ^b^	∨	∨	-
Perineural invasion	∨	-	-	-
Regional lymph nodes involved	M ^c^ > 10	5 < M < 10	0	0

^a^: the extent of metastatic potential of CCA indicated by No. of plus, the more plus indicate higher metastatic potential. ^b^: ∨ represent the presence of indicated metastatic index. ^c^: M represents the numbers of lymph nodes with the invasion of tumor.

## Data Availability

Not applicable, there is not any data available for supporting the results in this study.
